# Estimating canine cancer incidence: findings from a population-based tumour registry in northwestern Italy

**DOI:** 10.1186/s12917-017-1126-0

**Published:** 2017-06-28

**Authors:** Elisa Baioni, Eugenio Scanziani, Maria Claudia Vincenti, Mauro Leschiera, Elena Bozzetta, Marzia Pezzolato, Rosanna Desiato, Silvia Bertolini, Cristiana Maurella, Giuseppe Ru

**Affiliations:** 1 Biostatistics, Epidemiology and Risk Analysis Unit, Istituto Zooprofillattico Sperimentale del Piemonte, Liguria e Valle d’Aosta, Via Bologna 148, 10154 Torino, Italy; 2 Department of Veterinary Science and Veterinary Public Health, Università degli Studi di Milano, Milan, Italy; 3 Azienda Sanitaria Locale Valle d’Aosta, Aosta, Italy; 4 Azienda Sanitaria Locale TO4, Ivrea, Turin, Italy; 5 Histopathology Unit, Istituto Zooprofillattico Sperimentale del Piemonte, Liguria e Valle d’Aosta, Torino, Italy

**Keywords:** Population-based cancer registry, Dog, Incidence, Tumours, Cancer epidemiology, Sentinel animal

## Abstract

**Background:**

Canine cancer registry data can be put to good use in epidemiological studies. Quantitative comparison of tumour types may reveal unusual cancer frequencies, providing directions for research and generation of hypotheses of cancer causation in a specific area, and suggest leads for identifying risk factors. Here we report canine cancer incidence rates calculated from a population-based registry in an area without any known specific environmental hazard.

**Results:**

In its 90 months of operation from 2001 to 2008 (the observation period in this study), the population-based Piedmont Canine Cancer Registry collected data on 1175 tumours confirmed by histopathological diagnosis. The incidence rate was 804 per 100,000 dog-years for malignant tumours and 897 per 100,000 dog-years for benign tumours. Higher rates for all cancers were observed in purebred dogs, particularly in Yorkshire terrier and Boxer. The most prevalent malignant neoplasms were cutaneous mastocytoma and hemangiopericytoma, and mammary gland complex carcinoma and simplex carcinoma.

**Conclusions:**

The Piedmont canine cancer registry is one of few of its kind whose operations have been consistently supported by long-term public funding. The registry-based cancer incidence rates were estimated with particular attention to the validity of data collection, thus minimizing the potential for bias. The findings on cancer incidence rates may provide a reliable reference for comparison studies. Researches conducted on dogs, used as sentinels for community exposure to environmental carcinogens, can be useful to detect excess risks in the incidence of malignant tumours in the human population.

## Background

Canine cancer registry data can be put to good use in epidemiological studies. Quantitative comparison of tumour types may reveal unusual cancer frequencies, providing direction for research and generation of hypotheses of cancer causation in a specific area, and suggest leads for identifying risk factors.

The pivotal role of the sentinel animal some species play at a low level in the trophic chain, e.g., to show the effect of endocrine disruptors, [1] could also be assumed by dogs. The existence of a tumour registry in this species can pinpoint variations in cancer incidence in target organs. One task of such registries is to determine whether potential correlations exist between an increase or decrease in cancer incidence and environmental hazards. In a study conducted 25 years ago on companion animals as a sentinel for environmentally related human diseases, a correlation was found whereby changes in the canine proportionate incidence ratios preceded human incidence rates by 2 years, suggesting that fluctuations in proportionate incidence ratios of tumours in dogs may be useful for predicting changes in cancer patterns in humans [2]. Because these variations can only be discerned when the size of an observed population is known, and knowledge of the animal population size of a specific area is required for estimating the incidence of zoonotic diseases [3], veterinary epidemiologists face numerous hurdles to arriving at a correct estimate of an animal population at risk.

To get around this problem, some registries have obtained the denominator from pet insurance company databases [4, 5], but with the risk of having an incomplete denominator and introducing a systematic error. Insured dogs likely represent a selected subset of the real composition of an area’s dog population. [6]. Recognizing this limitation, veterinary studies have underscored the importance of selecting a delimited geographic area as referential for case collection when establishing animal population-based tumour registries. After an area has been selected, a distinction is made between cases belonging to the area and those not belonging to it. Cases belonging to the area but diagnosed outside of it then need to be identified and retrieved, and cases of animals living outside of it need to be excluded. It is acknowledged that the owners of dogs with cancer will often use veterinary services whereby cases are detected and reported, ultimately restricting the denominator to dogs receiving veterinary care.

The first population-based canine tumour registry was set up by the California State Department of Public Health to estimate the cancer incidence in dogs and cats resident in two counties in California, and to measure the effects of age, sex and breed on cancer development [7]. Over a 3-year period, malignant neoplasm cases in dogs and cats were reported to the registry through the collaboration of county veterinarians. The cases were categorized by their primary tumour site according to the International Classification of Diseases, Revision 7 (ICD-7). The estimated annual incidence rates for malignant cancers of all anatomic sites were 381.2/100,000 dogs and 155.8/100,000 cats.

Ten years later, MacVean et al. [8] investigated the denominator for the cancer incidence rate for dogs seen by veterinarians in one year in two counties. Histopathological diagnosis was offered free to the practitioners participating in the registry. The annual incidence rate for malignant neoplasms was 507/100,000 dogs, or double that of the Californian registry. Other European studies, using as denominator the cynological organization databases, reported incidence rates for some specific breeds [9–11].

The results of two cancer registration projects in Italy have recently been published. The Animal Tumour Registry of Genoa estimated an incidence rate for malignant cancers of 185.7/100,000 dogs [12]. The Animal Tumour Registry of Venice and Vicenza counties was set up in 2005 and provides free histopathological testing to veterinary practices in its catchment area. The estimated incidence rate for malignant neoplasms was 142.8/100,000 dogs [13].

Here we report canine cancer incidence data collected between 2001 and 2008 for a small, well-delimited geographical area in northwestern Italy. As the data were obtained from a population-based registry, the Piedmont Canine Cancer Registry, for an area without any known specific environmental hazard, they may be considered a reliable estimate of reference incidence rates and so may serve as comparison data for other registries.

## Methods

### Denominator

The catchment area was selected according to the following criteria: 1) a population of less than 20,000 dog units with an annually expected number of suspected cases bearable by the histopathological laboratory (the bearable burden of diagnostic testing was calculated using as “expected incidence” the figures reported in Dorn et al. [7] in their population-based registry in California); 2) both urban and rural environments to allow internal comparisons within the dataset; 3) a geographically well-delimited area.

**Fig. 1:**
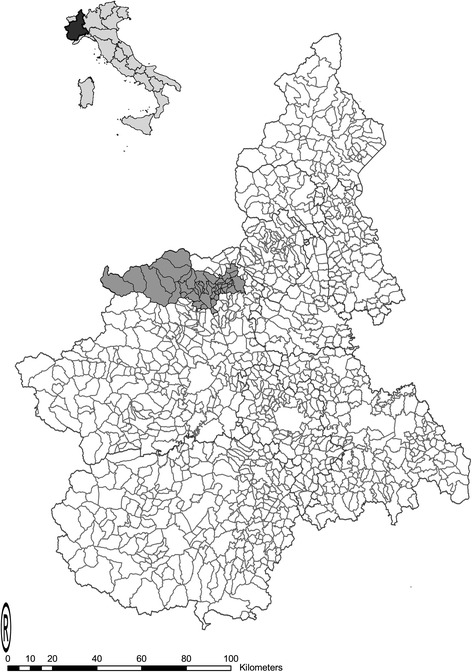
The catchment area of the canine tumour registry. *Left angle*: map of Italy and Piedmont; *Right angle*: map of Piedmont and the catchment area

Based on the above criteria, an area (Fig. 1) comprising 46 municipalities in northernwestern Piedmont was selected, where 17,770 dogs were recorded in the canine identification and registration system, which at the time (2001) was still in hard copy format. The area is under the administration of a single local health unit. It is bordered to the west and north by geographical barriers (Alps); on its southern and eastern borders it is surrounded by municipalities within the same local health unit that acted as a buffer area for data collection. The 22 veterinary practitioners in the catchment area and the buffer area were involved in the collection and identification of suspected tumours. The ArcGIS version 9.2 (ESRI, Redlands, CA, USA) was used to create the thematic map.

Surveys were carried out to estimate the real size of the dog population, removing the deceased subjects and including the unregistered dogs within the local canine identification and registration systems.

A capture-recapture (CR) method (Lincoln Petersen) [14] - normally used in ecology - was adopted and applied to the canine population of the catchment area. The CR method has been already applied to define the reference animal population in cancer registries [13] and is routinely used in epidemiological studies. The first stage of the method is the capture of a number of individuals (M), marked, and subsequently released within the general population.

In a second stage, a new random sample (n) is captured, out of which (m) is found to be already marked. If the marked subjects perfectly merge with the unmarked animals, the proportion of the marked individuals within the unknown overall population N and within the second sample n remains constant: m / n = M / N.

In this study, the first ‘capture’ is represented by the data from the regional canine identification and registration system, corrected after excluding the deceased subjects. This preliminary correction was carried out on the basis of a pilot telephone survey of the dog owners. The sample size of the survey (n = 545) was calculated on the basis of an expected prevalence rate of 15% of deceased dogs, an error of 3%, a 95% confidence interval (CI), and an expected response rate of 80%. The sample was stratified according to the number of dogs registered per municipality. The second random sample (recapture) was obtained by means of an anonymous questionnaire survey to estimate the proportion of unregistered dogs and, therefore, the overall population; the sample size was calculated using the same parameters (expected prevalence 15%, accepted error 3%, 95% CI). Moreover, the second survey was also used to estimate the structure of the canine population by age, sex, and breed and to estimate the proportion of owned dogs receiving veterinary care. The latter data were needed to estimate the proportion of the dog population that was actually seen in veterinary practices and could provide the incident cases to the laboratory. In urban areas the questionnaires were distributed in public offices (exam reservation centres), whereas in rural areas through the collaboration of the post office services which served also as questionnaire collection sites: 2000 hard copies of the questionnaire were printed out and distributed.

Finally, a dog population census based on 28,068 questionnaires sent to each household and covering most (44 out of 46) of the involved municipalities was conducted in 2005, four years after the start of the registration, by the local official veterinary service and used to validate and update the capture-recapture estimates.

### Numerator

A parallel public awareness campaign was launched through announcements in local print and media channels to inform dog owners about the canine tumour registry and its services. The tumour samples collected by biopsy, post-mortem or surgical removal were sent to the two diagnostic laboratories participating in the project and analysed free of charge. In addition, a dissection room was set up by the official veterinary service of the area for post-mortem examination and cancer detection if the owner decided to have the dog euthanised.

All veterinary practices were provided with a standardised case report form specifically designed for the collection of canine cancer cases. Because very few dogs had a subcutaneous microchip carrying their identification number, the animal’s name, breed, date of birth, sex, and the owner’s surname and address of the place of residence were recorded to identify each animal. Where the breed was not clearly indicated or two breeds were reported as indication of a mixed breed, this was included in the category “crossbreed”. Tumour data included anatomic site, lesion size, date of excision, and any related historical and clinical information (e.g., ovariohysterectomy or castration status). The participating practitioners were also provided with a form to report cases in which cancer diagnosis had been obtained from laboratories other than the two project laboratories.

Formalin-fixed samples were routinely processed, paraffin-embedded and stained with haematoxylin and eosin for histological examination. Immunohistochemical analysis was performed to characterise poorly differentiated neoplasms. Tumours were classified and coded according to the Classification of tumours of domestic animals, World Health Organization Tumor Fascicles and the International Classification of Diseases for Oncology, 3rd edition (ICD-0) [15–17].

Based on the histopathological diagnosis, each type of neoplasm of a multiple primary tumours was classified as a separate cancer and added to the numerator

### Cancer incidence calculation

A database (Microsoft Access) was created for collecting all cancer cases: information about sex, age, place of residence, veterinarian, number of tumours in the same dog, tumour classification, and tumour behaviour were entered into the database.

Incidence rates were calculated for histologically assessed malignant and benign tumours. In particular, specific rates by age, breed and sex were estimated with 95% confidence intervals. The incidence rates were expressed as the number of cases per 100,000 dog-years. A rate ratio was calculated to compare two subsequent registration period and its 95% confidence level was calculated to detect an eventual statistical significant difference (i.e. in case the 95% confidence interval would not include a value of 1). Statistical data analysis was performed using STATA software SE 11 (Stata Corp., College Station, TX, USA).

## Results

### Dog population size and structure

When the registration activity started in 2001, the reference canine population was estimated through a capture-recapture approach that yielded an overall population of 10,095 dogs (95% confidence interval (CI) 9705–10,485). As the first capture was based on dogs registered with the local identification and registration systems, 590 telephone interviews with dog owners were conducted (87% response rate) to preliminarily estimate the proportion of deceased dogs still registered. About half (55%) of the registered dogs had already died; therefore, the number of dogs registered and still alive (first capture) was estimated to be 8005. The second capture was obtained through an anonymous questionnaire survey (31.35% response rate) that yielded the identification of 627 dogs, 497 of which (79.3%) were recaptured as already registered with the canine registry office (95% CI 75.8–82.3). A 2005 midterm census of the municipalities in the catchment area (44 out of 46) confirmed the previous population estimates: the canine population was 9987 dogs as compared to the 9097 dogs estimated in the same 44 municipalities with the capture-recapture method (with a potential underestimation of 9.6%). Owing to the incompleteness of the census data (one of the two municipalities excluded was the largest town in the area), only the capture-recapture estimates were used as denominators for computing the incidence rates.

**Fig. 2:**
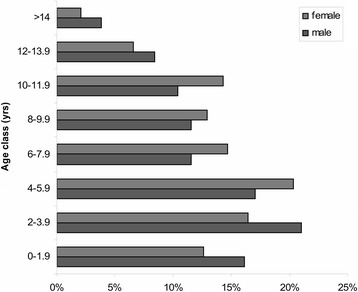
Population age structure by sex. Age structure of the study population, by sex

In the second survey, 91% of dog owners reported that they regularly used veterinary services for their dog’s health care (95% CI 88–93), suggesting that the veterinary practitioners were able to find tumours in 9182 dogs out of the 10,095 calculated. Based on the population size and the observation period (90 months), the total dog-years of observation to calculate the incidence rates were 68,865. In all, 55% of the dogs were male (95% CI 51–59%) and 48% were purebred (95% CI 44–52%), with 23 pure breeds (mainly German shepherd dog, Boxer, Yorkshire terrier, English setter and Siberian husky). Figure 2 shows the population structure by age.

### Numerators and incidence rates

**Fig. 3:**
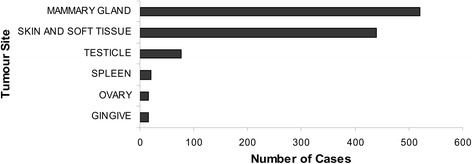
Number of cases by tumour site. Number of tumours collected during the study period, by tumour site

In its 90 months of operation, the Piedmont Canine Cancer Registry collected data on 1172 tumours, of which 618 benign and 554 malignant tumours were confirmed by histopathological diagnosis. Figure 3 presents the distribution of cases by most frequent tumour site.

The crude incidence rate was 804 per 100,000 dog-years for malignant tumours and 897 per 100,000 dog-years for benign tumours. As there was a decreasing trend for cancer cases at the end of 2002, we also calculated the incidence rates after splitting the registration period into two parts, before and after 01 June 2003 (period 1 vs. period 2); no statistically significant difference in the rates of malignant tumours was found (rate ratio [RR] 1.09, 95% CI 0.89–1.31) (Table 1).

**Table 1:** Crude incidence rates

	Number of cases	Dog-years	IR	95% CI
Crude	554	68,865	804	739–874
Female	344	31,113	1106	992–1229
Male	209	37,748	554	481–634
Purebred	311	32,820	948	845–1059
Crossbreed	233	36,045	646	566–735
Period 1	151	17,599	858	727–1006
Period 2	403	51,266	786	711–867

Malignant tumour incidence rates by sex, breed, and calendar period (cases per 100,000 dog-years at risk). *CI* denotes confidence interval, *IR* incidence rate

Overall, the higher cancer incidence rates in the female dogs were largely due to the high number of mammary tumours.

Higher incidence rates for all cancers were observed in purebreds. The incidence rates by breed are presented in Table 2. The most common breeds in the study population were the German shepherd dog (7785 dog-years), the Italian segugio (2003 dog-years), the English setter (1335 dog-years), and the Maremma sheepdog (1335 dog-years).

**Table 2:** Breeds

Breed	Number of cases	Dog-years	IR	95% CI
Yorkshire terrier	19	555	3423	2061–5346
Boxer	34	1223	2781	1925–3885
Alaskan malamute	3	113	2667	548–7759
Chow chow	3	113	2667	548–7759
Pinscher	7	443	1582	635–3256
Dalmatian	5	338	1481	480–3452
English cocker spaniel	9	668	1348	616–2558
Italian pointer	3	225	1333	275–3897
Siberian husky	14	1223	1145	626–1921
Rottweiler	10	893	1120	537–2059
English setter	13	1335	974	519–1665
German shepherd dog	71	7785	912	712–1150

Malignant tumour incidence rates in the most common breeds in the study population (cases per 100,000 dog-years at risk). *CI* denotes confidence interval, *IR* incidence rate. Breeds with incidence rates below 900 cases per 100,000 dog-years at risk not shown

**Fig. 4:**
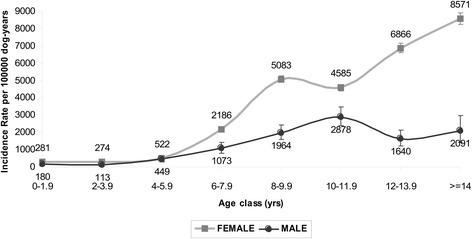
Age and sex-specific incidence rates. Age and sex specific incidence rates: cases per 100,000 dog-years at risk

**Fig. 5:**
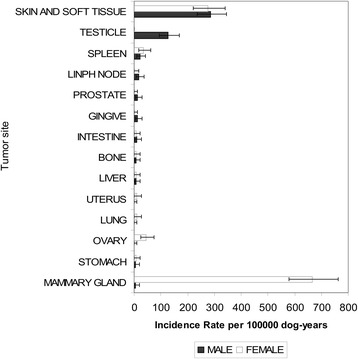
Malignant tumours: site- and sex-specific incidence rates. Site and sex specific incidence rates for malignant tumours: cases per 100,000 dog-years at risk

The cancer rates by age class in male and female dogs were similar up to the biennial class of 4–5.9 years, after which the rates started to diverge, with an increase noted for the females and a decrease for the males after the 10–11.9 age class (Fig. 4). The distribution of malignant tumours by sex and tumour site is shown in Fig. 5.

The most commonly affected organs were the mammary gland (*n* = 585), skin (*n* = 229), and ovaries (*n* = 40) in the female dogs and the skin (*n* = 242), testicles (*n* = 112), and spleen in the male dogs.

Table 3 reports the incidence rates of tumours by histological type and organ of origin (cases per 100,000 dog-years at risk) in the most frequently affected sites.

**Table 3:** Tumour site and histological type

Site	Tumour	ICD-O code	Number of cases	Dog-years	IR	95% CI
Mammary gland	Complex Carcinoma	8010/3	60	31,113	193	147–248
	Carcinoma Simplex	8231/3	36	31,113	116	81–160
	Tubulopapillary Carcinoma	8263/3	18	31,113	58	34–91
	Carcinoma In Situ	8010/2	18	31,113	58	34–91
	Solid Carcinoma	8230/3	16	31,113	51	29–84
Skin	Mastocytoma	9740/3	73	68,865	106	83–133
	Hemangiopericytoma	9150/1	28	68,865	41	27–59
Genital tract	Seminoma	9061/3	25	37,748	66	43–98
	Sertoli Cell Tumour	8640/1	13	37,748	34	18–59
Spleen	Hemangiosarcoma	9120/3	13	68,865	19	10–32

Incidence rates of malignant tumours (cases per 100,000 dog-years at risk) by histological type and site (only the higher incidence rates are shown). *CI* denotes confidence interval, *IR* incidence rate *ICD-O* International Classification of Diseases for Oncology

## Discussion

Here we present the findings obtained from a canine population-based cancer registry that we conducted in northwestern Italy for a 90-month period from 2001 to 2008. The Piedmont canine cancer registry is one of few of its kind whose operations have been consistently supported by long-term public funding. The incidence rate of malignant and benign tumours was about 800 and 900 cases per 100,000 dog-years at risk, respectively, with the largest impact on females and purebred dogs. The highest incidence rates by sex and tumour site were observed for mammary and cutaneous tissues in females and cutaneous tissues and testicles in males. These results were obtained by paying particular attention to the validity of the data collection process, thus minimizing the potential for bias.

Given the constraints derived from working with a canine population, considerable effort was expended to estimate the relevant denominators in terms of population size and structure. Moreover, we were able to obtain detailed reports of cases of incidental tumours by selecting a small-medium size catchment area and through public awareness campaigns coupled with enhanced participation of veterinary practices offered scientific support and histological examinations free of charge.

Though our study may have limitations, they do not necessarily compromise its internal or external validity. By mobilizing the available human and economic resources and restricting the cancer registration to a relatively small area (canine population of about 10,000 units), we were able to maintain the activities for a 90-month period, with a total of about 70,000 dog-years at risk which were then used as the overall denominator. The resulting overall incidence rates were found to be stable; however, less precise estimates could also have been obtained by looking at subgroups (e.g., municipality, breed, age class). For this reason, we carried out few subgroups analyses within our dataset.

As mentioned, there remains the potential for some residual bias in the quantification of the denominators and the numerators. Selection bias is less likely to be a problem with population-based registries than, for example, hospital-based registries [18, 19] or canine registries using databases from pet insurance companies where cross-breed dogs or aged dogs are apt to be underrepresented [6, 20].

With regard to the capture-recapture strategy that was applied to estimate the unknown canine population size, some of the assumptions this approach [14] requires may not have been fully met, e.g., working on a closed population and using random samples (assumptions that are unlikely to be met in a field situation); however, there are no evident reasons that preclude that the recruited dogs were equally likely to be captured in each of the two samples. The midterm census of 44 of the 46 municipalities in the catchment area indicated no large deviation from the direct enumeration of the existent dogs.

Additionally the population structure by breed- or age-strata may not be as precise as the population size. The structure was obtained from our second capture (the anonymous questionnaire survey) only. Unfortunately, the data on the first capture (i.e., the canine identification and registration system) and the midterm census were available only in hard copy format, precluding any practical way to further analyse the data. Therefore, the incidence rates are likely to be more robust when the denominator was the entire population (e.g., crude malignant rate or site-specific rates) rather than when the denominators referred to individual breeds or age classes.

Finally, with regard to the cases of incidental tumours, a certain degree of underdetection may have occurred. In particular, a proportion of tumours may have gone undiagnosed (e.g., a deep organ tumour), or not reported as not requiring histological confirmation (e.g., osteosarcoma, lymphoma) or may have been diagnosed by laboratories other than ours and not reported by the collaborating veterinary practitioners. In an attempt to minimize these problems, the collaborating veterinary practitioners were given standardized case report forms and local laboratories were contacted.

Assuming an unbiased estimation of cancer occurrence, the incidence rates in our registry are higher than those reported by similar population-based registries in Italy [12, 13] where the crude incidence rates for all malignant cancer were less than 200 cases per 100,000 dog-years at risk. The differences, albeit evident, are smaller when the comparison is carried out with data from international population-based registries ([7, 8, 21]). Rates higher than ours were reported in a registry that used as a denominator for cancer incidence all dogs insured with a pet U.K. insurance company [4]. The higher cancer rates we found cannot be convincingly linked to exposure of the dogs to as yet undetected environmental risk factors in the catchment area. Instead, the excess incidence may more likely stem from the effect of a different distribution of confounding factors such as age and breed, which are known determinants of cancer in dogs [22–24]. Unfortunately, in most of the comparable studies, data on the population structure by age and breed were not available, preventing any appropriate comparison of rates. Moreover unlike other studies, in ours the denominators were reduced to take into account the real proportion of dogs under veterinary care, an adjustment not reported elsewhere.

Another explanation for the high cancer rates we found may be due to the way we managed the data on multiple primary tumours, which are a common finding in dogs [25–27]. Based on the histopathological diagnosis, each type of neoplasm was classified as a separate cancer and added to the numerator. Other canine registries may not have done this. Finally, the potential risk of overestimation due to the inclusion of cases sourced from outside the registry catchment area was minimized by applying the preliminary case inclusion/exclusion criteria established in the registration protocol.

The distribution of cancer types in our registry is fairly consistent with the literature. As reported elsewhere [7–13, 21], the most prevalent malignant neoplasms were mammary carcinoma and cutaneous mastocytoma. The high prevalence of seminoma found in the Norwegian dog population [22] is analogous to the high incidence estimated in our registry. With regard to breed, the higher incidence rates in purebred rather than in cross-breed dogs were evident for Yorkshire terrier and Boxer, breeds known [5, 7, 27] to be particularly at risk of developing neoplasms, for which a genetic predisposition has been suggested. In the female dogs, the observed cancer trend by age matches those seen in other studies, with an exponential increase in elderly females for all tumours [9] or specifically for mammary tumours [5].

Comparison with the recent findings from two other Italian cancer registries [12, 13] shows that the distribution of tumours is substantially similar. In our registry, the ratio of malignant to benign tumours was 0.90:1, as compared to 0.96:1 in Genoa and 1:1 in the Veneto Region. The incidence of malignant cancer was higher in females than in males (2.5 times, 2.7 times, and 1.7 times in the study area, Genoa, and Veneto, respectively) and in purebred dogs than in cross-breed dogs (1.5 times in Piedmont and 2 times in Veneto where data were available). Finally, the distribution by tumour site (mammary and cutaneous/soft tissues tumours in females and cutaneous/soft tissues and genital tract tumours in males) was quite similar for all three registries. The exceptionally higher incidence of lymphoma in dogs of both sexes in the Genoa registry may have been due to the inclusion of cancers diagnosed by cytology or because of exposure to unknown risk factors present in a large city.

The Piedmont canine cancer registry is based on a large target population that combine rural and urban environments and on a population-based study design; for this reason our findings have the necessary external validity to be generalized, after adjustment for breed and age, to the canine population of northwestern Italy.

Assuming that the incidence rates from a population-based registry have external validity, national and international standards should be developed and shared to be able to make any meaningful comparison across studies. The adoption of an international classification and coding system, like the International Classification of Diseases for Oncology used in human cancer registries [28], would facilitate the exchange of comparable data and the possibility to carry out multicenter studies [29].

Finally, basic epidemiological methods (e.g., standardization techniques) to account for the confounding effect of age and breed should always be applied: data on population structure should be collected and made available in each study and, as proposed by Thrusfield [30], an international normal dog population should be established and shared as an external standard.

## Conclusions

As highlighted by Kelsey et al. [19], the data from population-based canine cancer registries may facilitate the identification of a less select group of cases for case-control studies and allow examination of trends over time and geographic differences in cancer incidence. The findings from the current study provide data on the incidence of canine tumours. The incidence rates may be useful for assessing the impact of neoplastic diseases in the canine population in northwestern Italy and may serve as a reference when setting up studies to detect excess risks in the incidence of malignant tumours in dogs used as sentinels for community exposure to environmental carcinogens.
